# Erector Spinae Plane Block as the Phrenic Nerve Sparing Anaesthetic Technique for Shoulder Arthroplasty

**DOI:** 10.7759/cureus.41220

**Published:** 2023-06-30

**Authors:** Fahad Farooq, Wojciech T Wierzejski

**Affiliations:** 1 Department of Internal Medicine, State University of New York Upstate Medical University, Syracuse, USA; 2 Graduate School of Medicine, University of Wollongong, Wollongong, AUS; 3 Department of Anaesthesiology, Broken Hill Base Hospital, Broken Hill, AUS

**Keywords:** shoulder replacement, erector spinal block, phrenic nerve palsy, post-operative pain management, brachial plexus block, total shoulder arthoplasty

## Abstract

The utilization of the brachial plexus block has become commonplace in shoulder replacement surgery and the management of postoperative pain. Nonetheless, this technique carries risks, including the occurrence of phrenic nerve palsy and subsequent postoperative dyspnea. In light of these concerns, the erector spinae plane block emerges as a safe, simple, and effective alternative for shoulder surgery with reduced risk of phrenic nerve palsy and potential motor sparing in the affected limb. This research endeavors to elucidate the analgesic application of erector spinae plane block (ESPB) through the presentation and analysis of two cases involving reverse shoulder arthroplasty.

## Introduction

The erector spinae plane block (ESPB) is a relatively new technique used for a wide variety of surgical analgesia, including acute and chronic pain. Forero et al. first documented the use of this block in 2016 for thoracic neuropathic pain, but it has since proven to be effective in use for a variety of procedures like hip, abdominal, and knee surgery [1-4].

In the context of shoulder surgery, the most common regional techniques of branchial plexus block (BPB) are supraclavicular and interscalene approaches, both of which pose a significant risk of phrenic nerve palsy, semi-diaphragmatic paralysis, and subsequent respiratory failure [5]. This is especially true in patients with a high risk of respiratory complications, such as those with chronic respiratory compromise, and in the obese [6-8]. ESPB subverts this risk while also providing good analgesia at multiple dermatome levels [1].

In this report, we describe two cases of patients undergoing total shoulder arthroplasty under continuous ESPB and general anesthesia (GA). We demonstrate the degree of phrenic nerve sparing by pre- and post-block peak expiratory flow (PEF) readings and ultrasound assessment of diaphragmatic excursion.

Prior to the procedure, both patients provided voluntary, informed consent for the ESPB as well as consent for publication.

## Case presentation

Case 1

**Figure 1:**
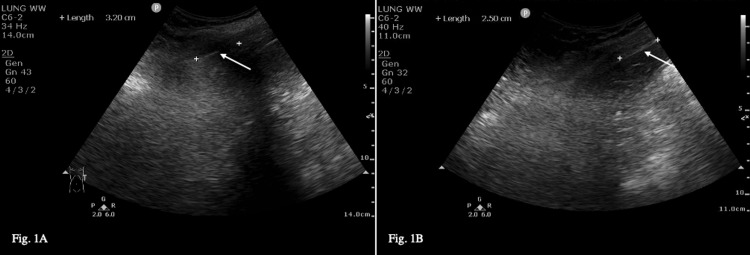
Pre-block (A) and post-block (B) diaphragm ultrasound for case 1.

An 84-year-old man (81 kg, BMI 29, ASA class 2) was admitted for left reverse shoulder replacement surgery after a traumatic injury to his shoulder approximately two years prior. Past medical history consisted of well-controlled hypertension, reflux disease, hyperlipidemia, right carotid artery stenosis, and prostate hypertrophy. His blood results, ECG, and other preoperative investigations were unremarkable. Given the patient's risk for postoperative respiratory complications secondary to Rockwood Clinical Frailty Scale 5, we discussed performing an ESPB. We obtained three pre-block peak expiratory flow rate (PEFR) values of 350, 450, and 470 L/min (Table 1). A pre-block diaphragm ultrasound was obtained by a senior anesthetist trained in point-of-care ultrasound and showed a diaphragmatic excursion of 3.20 cm (Figure 1A). With an aseptic technique and the patient positioned laterally, the ESPB was performed using a 21G SonoPlex (PAJUNK, Germany) needle and a high-frequency linear transducer (4-12 MHz, Sparq; Phillips, Andover, MA, USA) at the T1 transverse process. We injected 20 mL of 0.6% Ropivacaine in the erector spinae plane and inserted an 18G e-cath catheter (PAJUNK, Germany). The post-block PEFRs, taken 30 minutes later, were 350, 450, and 470 L/min (Table 1). The post-block diaphragm ultrasound showed a diaphragmatic excursion of 2.50 cm (Figure 1B). GA with endotracheal intubation (ETT) was started after the block, and surgery was performed in a reclined beach chair position with standard monitoring as required for this procedure. The surgery lasted 120 minutes, and there were no complications. During the procedure, the patient received 989 μg of Remifentanil IV and 90 mg of Clonidine IV. The patient’s verbal pain score in the recovery room was <2/10. We started an infusion of 0.2% Ropivacaine at 5 mL/hour four hours after surgery and six hours after the initial block. Neurovascular observations of the upper limbs, performed by senior nursing staff, were found to be normal 10 hours after surgery. The 0.2% Ropivacaine infusion was continued for four days, for a cumulative total of 346 mL during this time. The patient also received a Fentanyl PCA with 10 μg/bolus and a five-minute lockout time for one day, cumulatively receiving 150 μg. His respiratory rate remained between 15 and 20 breaths/min, and his verbal pain score remained <4/10 until discharge four days post-procedure.

Case 2

**Figure 2:**
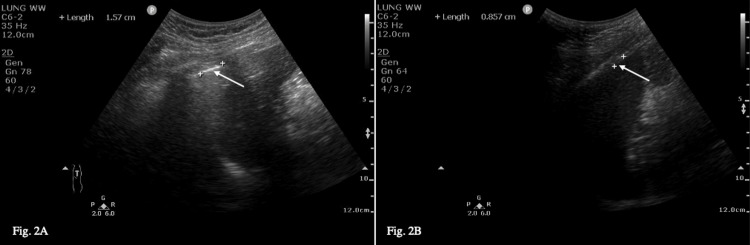
Pre-block (A) and post-block (B) diaphragm ultrasound for case 2.

An 82-year-old woman (91 kg, BMI 35, ASA class 3) with a three-year history of disabling left shoulder pain secondary to degenerative joint disease was admitted for left reverse shoulder replacement surgery. Her past medical history included obesity class 2, hypertension, hiatus hernia, diverticular disease, and type 2 diabetes. She had no significant derangements in blood results, ECG, or other pre-operative investigations. Given the high BMI of the patient, the Rockwood Clinical Frailty Scale 5, and the risks of postoperative respiratory complications, the option for ESPB was accepted. We obtained three pre-block PEFRs of 240, 250, and 260 L/min (Table 1). A pre-block diaphragm ultrasound showed a diaphragmatic excursion of 1.57 cm (Figure 2A). With an aseptic technique and the patient positioned laterally, the ESPB was performed using a 16G Tuohy needle (PORTEX, Czech Republic) and a high-frequency linear transducer (4-12 MHz, Sparq; Phillips, Andover, MA, USA) at the T2 transverse process. We injected 20 mL of 0.5% Ropivacaine in the erector spinae plane and inserted a 16-G multi-orifice catheter (PORTEX, Czech Republic). The post-block PEFRs, taken 30 minutes later, were 220, 250, and 260 L/min (Table 1). Post-block diaphragm excursion was 0.86 cm on ultrasound (Figure 2B). Surgery was performed under GA with ETT in a reclined beach chair position with standard monitoring and lasted 140 minutes without complications. During the procedure, the patient received 642 μg of IV Remifentanil and 1 g of IV Paracetamol. The patient’s verbal pain score in the recovery room was <3/10. We started an infusion of 0.2% Ropivacaine at 5 mL/hr one hour after surgery and four hours after the initial block. Neurovascular observations of the upper limbs, performed by senior nursing staff, were found to be normal eight hours after surgery. The 0.2% Ropivacaine infusion was continued for two days, for a cumulative total of 213 mL during this time. The patient also received a Fentanyl PCA of 10 μg/bolus with a five-minute lockout time for one day, cumulatively receiving 87 μg over this period. Her respiratory rate remained between 15 and 20 breaths/min, and her verbal pain score remained <3/10 until discharge two days post-procedure.

**Table 1:** A comparison of pre- and post-block diaphragmatic excursion and peak expiratory flow rate. *This value represents the mean between three readings as described in the case.

	Diaphragmatic excursion		PEFR^*^	
	Pre-block	Post-block	Pre-block	Post-block
Case 1	3.20 cm	2.50 cm	423 L/min	423 L/min
Case 2	1.57 cm	0.86 cm	250 L/min	243 L/min

## Discussion

The ESPB is a relatively new technique in which local anesthetic is injected over the transverse process beneath the erector spinal muscles. It is commonly performed with in-plane ultrasound guidance, in which the whole needle is visualized and controlled throughout the procedure. In addition to single-shot analgesia, continuous infusion can be given via catheter for postoperative analgesia.

Though ESPB has been used in a variety of cases, we propose that it has a particularly good use case in shoulder surgery, where complications of BPB are not uncommon. The most notable yet accepted complication of BPB in this context is hemi-diaphragmatic paralysis resulting from local anesthetic going over the phrenic nerve [5,8], the incidence of which approaches 100% [9]. Phrenic nerve palsy, in turn, may lead to dyspnea and hypoxic episodes in high-risk patients such as the obese (BMI >30) [6,7] and those with pre-existing respiratory disease [8].

The main advantages of ESPB over BPB include simplified technique, a lower risk of respiratory complications, and potential motor function sparing. In this block, the needle touches the surface of the transverse process at the level of required analgesia. This is the natural limit of the propagation of the needle to more deep tissue layers and reduces the risk of complications like nerve injury or pneumothorax [10,11]. The local anesthetic infiltrates the dorsal rami of the spinal roots with minimal penetration to the ventral rami, which may explain the good sensory block with no or minimal motor blockade [12,13]. Based on the available literature about the motor-sparing effect of the ESPB, we decided to extend the regional anesthesia using the continuous infusion of Ropivacaine via catheter to mitigate the risk of postoperative delirium, where the use of opioids plays a prominent role.

Given the BMI and frailty of our cases, we recognized that postoperative respiratory management was a concern. As we attempted to describe phrenic nerve sparing with this block, we documented pre- and post-block PEFR and used diaphragmatic excursion as a surrogate for respiratory and hemi-diaphragmatic function. Low PEFR values are correlated with an increased incidence of postoperative respiratory complications [14,15]. We used a 2015 study by Scheibe et al. to guide our diaphragm ultrasound technique [16]. Our patients showed no evidence, either on observation, ultrasound, or PEFR values, of phrenic nerve dysfunction causing paralysis of the hemidiaphragm. A slight decrease in diaphragmatic excursion was acceptable given there were no postoperative complications or complaints of dyspnea, and there was no sign in either case of paradoxical diaphragmatic movement characteristic of diaphragmatic palsy. Given this benefit, our findings are in line with previous research suggesting this block is a viable alternative to BPB, particularly in a high-risk population.

Pain scores remained low in both cases. Average pain scores for postoperative analgesia have not been shown to be significantly different for patients receiving BPB versus ESPB one day after surgery [17]. Subjectively, we did not notice a difference in analgesia consumption in these patients versus those conventionally receiving BPB.

## Conclusions

In conclusion, the ESPB demonstrates favorable attributes as a safe, simple, and effective technique for achieving operative and postoperative analgesia in the context of shoulder arthroplasty. It is important to acknowledge the limitations of our case series. First, it should be noted that the PEFR is utilized as an indicator of expiratory muscle strength, albeit offering only an indirect assessment of the inspiratory force generated by the diaphragm. Thus, it provides an incomplete reflection of the end-inspiratory volume, an important metric for evaluating diaphragmatic function. Second, a systematic evaluation of upper limb motor function was not conducted in a manner that would allow quantifiable comparisons for future research. Consequently, we recommend further investigations that compare the sensory and motor deficits associated with ESPB and BPB. Finally, while our case series serves as a successful report, caution should be exercised in extrapolating its findings to establish local or national surgical or anesthetic policies.
